# Gestational diabetes risk in a multi-ethnic population

**DOI:** 10.1007/s00592-019-01404-8

**Published:** 2019-09-07

**Authors:** Anat Jaffe, Shmuel Giveon, Carmit Rubin, Ilya Novikov, Arnona Ziv, Ofra Kalter-Leibovici

**Affiliations:** 1 Endocrinology and Diabetes Unit, Hillel Yaffe Medical Center, Hashalom St., 38100 Hadera, Israel; 2 Clalit Health Services, Tel Aviv, Israel; 3 Information and Computerization Unit, Gertner Institute for Epidemiology and Health Policy Research, Sheba Medical Center, Tel-Hashomer, Ramat Gan, Israel; 4 Unit of Cardiovascular Epidemiology, Gertner Institute for Epidemiology and Health Policy Research, Sheba Medical Center, Tel-Hashomer, Ramat Gan, Israel; 5 The School of Public Health, Sackler Faculty of Medicine, Tel-Aviv University, Tel-Aviv, Israel

**Keywords:** Body mass index (BMI), Diabetes in pregnancy, Ethnicity, Gestational diabetes mellitus, Metabolic syndrome

## Abstract

**Aims:**

To compare gestational diabetes mellitus (GDM) risk among two ethnic minority groups, with high type-2 diabetes (T2DM) prevalence, as compared to the Jewish population majority group.

**Methods:**

A historical cohort study was conducted using clinical data collected between January 1, 2007, and December 31, 2011. The study sample included 20–45-year-old women; 2938 Ethiopian, 5849 Arab and 5156 non-Ethiopian Jewish women. GDM was defined according to the two-step strategy: step 1: glucose ≥ 140 mg/dl and step 2: using Coustan and Carpenter’s diagnostic criteria. GDM risk was tested in a multivariable model, adjusted for age, parity and pre-gestational values of the metabolic syndrome components.

**Results:**

Mean body mass index (BMI) values and morbid obesity rates were lowest among Ethiopian women and highest among Arab women. The prevalence of pre-gestational diabetes was significantly higher among Ethiopian (2.7%) and Arab (4.1%) women than among non-Ethiopian Jewish women (1.6%), and GDM screening rates were relatively high (85.5%, 87.2% and 83%, respectively). The proportion of pregnancies complicated with GDM was higher among Ethiopian women (4.3%) but not significantly different between Arab (2.9%) and non-Ethiopian Jewish (2.2%) women. In multivariable analysis, GDM was associated with Ethiopian ancestry (OR, 2.55; 95% CI, 1.60–4.08), adjusted for age, BMI, plasma triglyceride level and parity. Arab ethnicity was not significantly associated with GDM risk in multivariable analysis.

**Conclusions:**

Both Ethiopian and Arab minority ethnicities have a higher risk of T2DM in comparison with other Israeli women, but only Ethiopian origin is an independent risk factor for GDM while Arab ethnicity is not.

## Introduction

Gestational diabetes mellitus (GDM) defined as glucose intolerance first diagnosed during pregnancy is associated with a higher risk of adverse obstetric and perinatal outcomes [1, 2]. Moreover, women with GDM have sevenfold greater risk of developing type-2 diabetes (T2DM) 5–10 years after delivery [3], and offspring of mothers with GDM have higher obesity and diabetes mellitus (DM) rates later in life [4, 5].

The prevalence of GDM and T2DM differs among ethnic minority groups [6, 7]. In the USA, Asian and Filipino women have higher prevalence of GDM and T2DM compared to non-Hispanic White women, while African-American women have higher prevalence of T2DM but not of GDM [8, 9].

Arabs are the largest ethnic minority group in Israel, accounting for 21% of the population [10]. Arab women have high prevalence of obesity and central obesity [11] and higher risk of T2DM compared to the Jewish female majority population [12].

Ethiopian Jews have immigrated to Israel since 1984 and account for 1.7% of the population [13]. On arrival to Israel, the prevalence of DM among Ethiopian Jews was less than 1% [14] and increased rapidly thereafter. Recent studies reported higher prevalence of DM among Ethiopian Jewish women at reproductive age and lower prevalence of obesity compared to the majority group of non-Ethiopian Jewish women in Israel [15, 16].

Data are lacking on the prevalence and risk factors for GDM among Arab and Ethiopian Jewish women. These data are pertinent for pre-pregnancy prevention and early detection of GDM and for timely treatment, and are thus the focus of the current study.

## Methods

A historical cohort study was conducted using clinical data collected between January 1, 2007, and December 31, 2011, in the electronic medical records of Clalit Health Services (CHS) database. The study sample included women who were 20–45 years old on January 1, 2008, residents of the mostly urban Sharon and Hadera districts in central Israel, and insured by CHS. The sample was stratified by ethnicity and included women of Ethiopian ancestry, Arab women and non-Ethiopian Jewish women. CHS is the largest health plan in Israel and insures more than 86% of Ethiopian Jews, 76% of Arabs and 46% of non-Ethiopian Jews in the two districts.

Data collected included demographics, laboratory test results, chronic medical therapy, hospital admissions and chronic diagnoses. We included information on live births between January 1, 2008, and December 31, 2011 (the study period).

For most women (98%), GDM diagnosis was based on a 2-step screening protocol of 50 g oral glucose challenge test (GCT) and 100 g 3-h oral glucose tolerance test (OGTT). GDM was defined by 1-h post-GCT plasma glucose (PG) ≥ 200 mg/dl, or 1-h post-GCT PG ≥ 140 mg/dl and < 200 mg/dl and at least two plasma glucose values equal or greater than the plasma glucose thresholds set by Carpenter and Coustan glucose thresholds in 3-h OGTT: fasting—95 mg/dl; 1 h—180 mg/dl; 2 h—155 mg/dl, or 3 h—140 mg/dl [17].

Pre-gestational diabetes was defined in non-pregnant women by physician’s diagnosis of DM, purchases of three or more hypoglycemic drug prescriptions, or at least two values of HbA1c, fasting or post-75 g oral glucose load plasma glucose within the DM range [18].

We included all births during the study period to calculate the proportion of pregnancies complicated with GDM, while only first births complicated with GDM were used for GDM risk analysis. Births among women with pre-gestational diabetes were excluded from the current analysis.

### Statistical analysis

Comparisons of baseline characteristics between non-Ethiopian Jewish women (the reference group) and women of the two ethnic minority groups were carried out, using appropriate contrasts in a mixed linear model for continuous variables, and the Chi-square test for discrete variables with Bonferroni correction for multiple comparisons. The total number of pregnancies in the three ethnic groups was compared using Poisson regression. Proportions of pregnancies screened for GDM were compared using repeated measured logistic regression.

The association between ethnicity and GDM risk was tested in multiple logistic regression analysis, adjusted for age, parity (number of children ≤ 18 year on 01/01/2008), whether it was a single or multiple pregnancy, and pre-gestational levels (for women who gave birth) or first values recorded during follow-up (for other women) of the metabolic syndrome components other than plasma glucose (i.e., fasting plasma triglycerides and HDL cholesterol, systolic blood pressure, and body mass index (BMI). Missing values were treated by multiple imputation approach.

The CHS institutional ethics committee approved the study protocol. In accordance with the Israeli Ministry of Health regulations, informed consent was not required because all identifying information had been removed from the study dataset.

## Results

### Characteristics of the study sample

The study sample included 13,943 women, of whom 5156 were non-Ethiopian Jews, 2938 were Ethiopian Jews and 5849 were Arabs. The mean age was 30 years and did not differ by ethnicity (Table 1). Compared to non-Ethiopian women, Arab women had higher (mean ± SD) BMI 27.0 ± 6.3 versus 24.7 ± 5.7 kg/m^2^ (*p* < 0.05); higher prevalence of morbid obesity (BMI ≥ 35 kg/m^2^): 9.9% versus 6.2% (*p* < 0.05); and higher prevalence of pre-gestational diabetes: 4.1% versus 1.6% (*p* < 0.05). Although the Ethiopian women compared to non-Ethiopian Jewish women had lower mean BMI values, (23.2 ± 4.6 vs. 24.7 ± 5.7 kg/m^2^) and lower morbid obesity rate [1.1% vs. 6.2% (*p* < 0.05)], the prevalence of pre-gestational diabetes was significantly higher [2.7% vs. 1.6% (*p* < 0.05)].

**Table 1:** Characteristics of the study cohort by ethnicity

	Non-Ethiopian Jews (reference)	Ethiopian Jews	Arabs
Number	5156	2938	5849
Age, years (mean ± SD)	30.9 ± 6.4	30.5 ± 7.2	30.0 ± 7.0
BMI kg/m^2^ (mean ± SD) (number)	24.7 ± 5.7 (3406)	23.2 ± 4.6 (1814)	27.0 ± 6.3* (4260)
BMI ≤ 22.5 kg/m^2^ (number, %)	1468 (43.1%)	916 (50.5%)*	1064 (25.0%)*
BMI ≥ 35 kg/m^2^ (number, %)	210 (6.2%)	19 (1.1%)*	422 (9.9%)*
HDL-C mg/dl (mean ± SD) (number in the category)	54.4 ± 13.3 (4236)	52.5 ± 12.0 (2425)	50.2 ± 11.5 (5077)
Triglycerides, mg/dl (mean ± SD) (number)	101.1 ± 57.9 (4347)	90.5 ± 53.1 (2483)	101.8 ± 67.9 (5157)
SBP mmHg (mean ± SD) (in the category)	112.8 ± 12.2 (4587)	114.0 ± 12.8 (2733)	115.1 ± 11.5 (5607)
Non-gestational diabetes (number, %)	84 (1.6%)	78 (2.7%)*	241 (4.1%)*
Women with ≥ 1 delivery^a^ (number, %)	1959 (38%)	844 (29%)	2369 (40.5%)
Total number of children ≤ 18 years	1.8 ± 1.5	1.9 ± 1.5*	2.2 ± 1.6*
Number of births per woman^a^	0.48	0.36*	0.53*
Multiparous^a^ (number, %)	3662/5156 = 71%	2293/2938 = 78%*	4138/5849 = 70.7%
Total screening for GDM (%)^b^	83.0%	85.5%	87.2%*
One-step screening for GDM	49/2416 = 2.0%	15/1017 = 1.5%	67/2997 = 2.2%
Proportion of pregnancies complicated with GDM (number, %)	53/2416 = 2.2%	44/1017 = 4.3%^*^	86/2997 = 2.9%

Values of BMI represent the last measurement before the first pregnancy or first recorded BMI for non-pregnant women during the study period. Other parameters represent the first measurement in the study period

*BMI* body mass index, *HDL-C* high-density lipoprotein cholesterol, *SBP* systolic blood pressure, *GDM* gestational diabetes mellitus

^a^Among women who delivered during the study period (including women with diabetes)

^b^Including one-step and two-step screening

*P < 0.05; compared to non-Ethiopian Jewish women—reference majority population

Compared to non-Ethiopian Jewish women, the number of births per woman during the study period was lower among Ethiopian women (0.36 vs. 0.48; (*p* < 0.001) and higher among Arab women (0.53, *p* = 0.001) (Table 1).

Arab women had the highest performance rate of GDM screening (87.2%) of the three ethnic groups, while the screening rates did not significantly differ between Ethiopian and non-Ethiopian Jewish women (85.5% and 83.0%, respectively) (Table 1).

Compared to non-Ethiopian Jewish women, the proportion of pregnancies complicated with GDM was higher among Ethiopian women (4.3% vs. 2.2%; *p* < 0.001) but did not differ significantly among Arab women (2.9%; *p* = 0.12).

### Characteristics of pregnant women by ethnicity

In all ethnic groups, women with GDM were older and had higher values of BMI, plasma triglycerides and systolic blood pressure (Table 2). Ethiopian Jewish woman with GDM were, on average, 3.5 years older than reference women with GDM and had significantly lower mean (± SD) BMI 25.0 ± 4 versus 30.6 ± 5 kg/m^2^, respectively.

**Table 2:** Characteristics of pregnant women with or without GDM by ethnicity

	Non-Ethiopian Jewish women (reference)		Ethiopian Jewish women		Arab women	
	NGT	GDM	NGT	GDM	NGT	GDM
Number	1590	50	673	43	1977	83
Age, years (mean ± SD)	30.3 ± 4	31.7 ± 5*	30.9 ± 5	35.1 ± 5*,**	27.9 ± 5	32.1 ± 6*
BMI kg/m^2^ (mean ± SD) (number)	23.8 ± 5 (654)	30.6 ± 5* (19)	22.4 ± 4 (248)	25.0 ± 4*,** (18)	25.2 ± 5 (951)	28.6 ± 5* (38)
HDL-C mg/dL (mean ± SD) (number)	57 ± 13 (880)	48 ± 12* (32)	54 ± 12 (384)	54 ± 11 (32)	52 ± 12 (1179)	52 ± 13 (56)
Triglycerides, mg/dL, (median (IQR)) (number)	80 (61,115) (968)	116* (84,151) (33)	75 (57, 101) (405)	99* (72, 143) (34)	79 (58, 117) (1249)	113* (76, 152) (57)
SBP mmHg (mean ± SD) (number)	109 ± 12 (764)	117 ± 10* (22)	109 ± 12 (372)	115 ± 11* (26)	112 ± 11 (1151)	116 ± 12* (51)

Values of age, BMI, HDL-C, triglycerides and SBP represent the last measurement before the first pregnancy during the study period

*GDM* gestational diabetes mellitus, *NGT* normal glucose tolerance,*BMI* body mass index, *HDL-C* high-density lipoprotein cholesterol,*SBP* systolic blood pressure

*Comparisons between women with and without GDM, within ethnic group *p* < 0.05

**Comparisons between Ethiopian Jewish or Arab women with GDM and non-Ethiopian Jewish women—reference majority population with GDM, *p* < 0.001

### GDM risk factors

Adjusted for age, BMI, parity and pre-pregnancy values of systolic blood pressure, plasma triglycerides and HDL cholesterol, Ethiopian ancestry was associated with higher likelihood for GDM [odds ratio (OR) 2.55; 95% confidence interval (CI) 1.6–4.1], while Arab ethnicity was not (OR 1.43; 95% CI 0.95–2.15 *p* = 0.087). Other factors associated with greater risk of GDM included older age, higher BMI and higher plasma triglycerides levels, while parity was associated with lower risk (Table 3). Systolic blood pressure and HDL cholesterol were not significantly associated with GDM. There were 82 live twin births. Further adjustment for multiple versus single pregnancy did not materially change the point estimates in the multivariable model (data not shown).

**Table 3:** Factors associated with GDM: multivariable analysis

Parameter	Odds ratio	95% Confidence limits		*P*
*Ethnic group*				
Non-Ethiopian Jewish women (reference category)	1.00	–	–	
Arab women	1.43	0.95	2.16	0.087
Ethiopian women	2.55	1.60	4.08	< 0.0001
Age (per 10 years increment)	2.92	2.09	4.07	< 0.0001
BMI	1.12	1.06	1.18	0.0007
HDL-C (per 5 mg/dl increment)	0.99	0.91	1.08	0.83
Triglycerides (per 10 mg/dl increment)	1.05	1.02	1.09	0.004
SBP (per 5 mm/Hg increment)	1.06	0.92	1.20	0.39
Parity	0.77	0.66	0.89	0.0006

* SBP* systolic blood pressure, *HDL-C* high-density lipoprotein cholesterol, *BMI* body mass index

## Discussion

We found that Ethiopian women had a 2.5-fold greater risk of GDM compared to non-Ethiopian Jewish women, independent of maternal age, body weight, blood pressure and dyslipidemia. In fact, Ethiopian women with GDM had significantly lower body weight compared to reference and Arab women with GDM. The higher risk of GDM among Ethiopian women is in line with recent studies showing high risk of adult-onset DM among Ethiopian Jews younger than 50 years of age, particularly women [15, 16, 19].

In our study, Arab ethnicity was not found to be significantly associated with a greater risk of GDM, although Arab women had higher prevalence of pre-gestational diabetes compared to non-Ethiopian Jewish women. Previous studies have shown that Arab men and women are at greater risk of T2DM [12, 20]. Possibly, this study was underpowered to show a smaller ethnic difference in GDM risk among Arab and non-Ethiopian Jewish women. Other studies examining ethnic minorities in the USA found that African-American women also have higher prevalence of T2DM and similar prevalence of GDM compared to non-Hispanic White women [9]. Lawrence et al. [21] suggested that the differences in the effect of ethnicity on GDM versus T2DM risk might be due to a higher proportion of ethnic minority women with pre-gestational diabetes, leaving a smaller fraction of the population at risk of GDM.

Ethnic disparities in GDM risk have been reported in other populations. Filipino and Asian women have a significantly higher prevalence of GDM and T2DM compared to non-Hispanic White Americans, even in normal and low BMI categories [9]. The mechanism for the different effect of BMI and ethnicity on GDM and T2DM risk is unclear. Differences in ethnic-related body composition and fat distribution have been suggested in the Multicultural Community Health Assessment Trial (M-CHAT) [22], Multi-Ethnic Study of Atherosclerosis (MESA) [23] and Mediators of Atherosclerosis in South Asians Living in America (MASALA) studies [24]. Other genetic factors that modulate insulin resistance and β cell function may also play a role. South Asians have higher values of insulin resistance and lower values of β-cell function than other ethnic groups (Chinese, African-American, Latino and non-Hispanic Whites) even after adjusting for age and adiposity [25].

Similar to other studies, we also found that age, BMI and plasma triglyceride levels were significantly associated with a higher risk of GDM [26–28]. In contrast to previous studies [29], we found no association between systolic blood pressure and GDM risk. This may be explained by imperfect standardization of blood pressure measurements performed and recorded in a clinical setting.

We found that parity was associated with lower risk of GDM, after controlling for the effect of age and BMI. Recently, Sweeting et al. [30] reported that parous women without a history of GDM had a lower risk of GDM in subsequent pregnancies. Seventy-two percent of the pregnant women in our study were multiparous. It is conceivable that women with a history of GDM before the study period were more likely to develop T2DM and were therefore excluded from this study.

The proportion of pregnancies complicated with GDM in our study (2.2% among non-Ethiopian Jewish women) was somewhat lower than previously reported in Israel. The proportion of pregnancies complicated with GDM reported by Sella et al. was 4.3% in a mostly Jewish population of women insured by the second largest health plan in Israel [28]. The higher GDM rates reported by Sella et al. compared to the current study may be partially explained by a higher proportion of women diagnosed with a one-step screening test, using 3-h 100 g OGTT (9% vs. 2%), women without a positive OGTT who initiated insulin treatment after GDM screening (9.8% vs. 0%) and a slightly older mean age (31.4 vs. 30.9 years, respectively) [31].

In the current study, GDM diagnosis was based on the two-step strategy, using the Carpenter and Coustan diagnostic criteria, which was the most common practice in Israel during the study period. These criteria are less sensitive compared to those of International Association of Diabetes in Pregnancy Study Groups/World Health Organization (IADPSG/WHO) [18]. The HAPO study showed that the association between maternal hyperglycemia and adverse maternal and fetal outcomes is continuous, without a clear-cut threshold [32, 33]. Thus, plasma glucose levels that are lower than the glucose cutoffs recommended by Carpenter–Coustan are still associated with significant risk of adverse maternal and fetal outcomes. Differences in GDM prevalence and in the relative diagnostic importance of fasting, 1-h and 2-h plasma glucose were observed across the ethnically diverse centers of the HAPO study [34]. Adopting the IADPSG/WHO diagnostic criteria for GDM is expected to significantly increase the number pregnancies diagnosed with GDM [35].

This study has few limitations: The analysis was based on data collected for clinical and administrative purposes. However, there is high utilization rate of prenatal care services in Israel (i.e., free access to family physicians, obstetric care and laboratory testing), and thus, underestimation of GDM prevalence is not likely. The statistical analyses were based on live births only and did not include stillbirths. Our database did not include information on GDM in previous pregnancies, family history of DM, socioeconomic status and lifestyle habits (diet and physical activity), all of which are significant risk factors for GDM.

Nevertheless, this is the first population-based study that provides epidemiological data on the proportion of pregnancies complicated with GDM and the risk determinants for GDM in two ethnic minority groups living in the same region: Ethiopian and Arab women, including comparisons with the majority non-Ethiopian Jewish population.

## Conclusions

We have shown that Ethiopian women are at greater risk of GDM despite having lower mean BMI. Special efforts should be directed toward prevention and early diagnosis of GDM among Ethiopian women to reduce maternal and fetal adverse outcomes associated with impaired glucose metabolism in pregnancy. Indeed, it was already suggested in the article “diabetes in pregnancy” [36]: the goal is to improve pregnancy outcomes in women with gestational diabetes through sustainable policies of screening and treatment.

Further research is needed to understand the higher susceptibility to GDM among Ethiopian women despite lower BMI.
